# Judgment Line Orientation Test: a proposal for an abbreviated version for the Brazilian population

**DOI:** 10.1002/alz70857_102769

**Published:** 2025-12-24

**Authors:** Ana Beatriz Silva, Maria Paula Foss, Flávia Lima Osório, Vitor Tumas

**Affiliations:** ^1^ Faculty of Philosophy, Sciences, and Letters of the University of São Paulo at Ribeirão Preto (FFCLRP‐USP), Ribeirão Preto, São Paulo, Brazil; ^2^ Clinical Hospital of the Ribeirão Preto Medical School of the University of São Paulo (HCFMRP‐USP), Ribeirão Preto, São Paulo, Brazil; ^3^ Faculdade de Medicina de Ribeirão Preto, Ribeirão Preto, São Paulo, Brazil

## Abstract

**Background:**

The Judgment Line Orientation (JLO) Test is a widely used neuropsychological tool for assessing visuospatial abilities. Its original 30‐item version is lengthy and fatiguing for elderly individuals, especially those with cognitive impairments. This study aimed to develop and adapt an abbreviated version of the JLO for the Brazilian elderly population using Item Response Theory (IRT) and following the International Test Commission (ITC) guidelines.

**Method:**

The study included 113 participants, divided equally into a clinical group (with Mild and Major Neurocognitive Disorders per DSM‐5 criteria) and a control group (cognitively unimpaired). Participants were assessed using the JLO, the Clinical Dementia Rating (CDR), the Wechsler Abbreviated Scale of Intelligence (WASI), and the Jaeger Visual Acuity Chart. IRT was employed to analyze and select the most effective items from the JLO, creating a shorter version.

**Result:**

The clinical group scored significantly lower than the control group, highlighting the impact of cognitive impairment on visuospatial performance. Male participants outperformed females by approximately 2 points, and better education correlated positively with higher scores. Significant correlations were observed between the JLO and WASI subtests, including Cubes (*r* = 0.68) and Matrix Reasoning (*r* = 0.71). Using IRT, a 6‐item version of the JLO was developed. The abbreviated version demonstrated a strong correlation with the original test (*r* = 0.96) while significantly reducing administration time, maintaining high reliability and diagnostic accuracy.

**Conclusion:**

The abbreviated JLO is a practical, culturally adapted tool for assessing visuospatial deficits in Brazilian elderly individuals. This shorter version is highly efficient and valid, facilitating the early detection of neurocognitive disorders in clinical and hospital contexts. Its application enhances diagnostic precision while reducing the burden on both patients and professionals.

